# Children wearing face masks to prevent communicable diseases: scoping review

**DOI:** 10.1590/1984-0462/2023/41/2021164

**Published:** 2022-07-15

**Authors:** Patrícia Pinto Braga, Meriele Sabrina de Souza, Patrícia Peres de Oliveira, Márcia Christina Caetano Romano, Gustavo Machado Rocha, Elaine Cristina Rodrigues Gesteira

**Affiliations:** a Universidade Federal de São João del-Rei, São João del-Rei, MG, Brazil.

**Keywords:** Child health, Respiratory protective devices, Disease transmission, infectious, Saúde da criança, Dispositivos de proteção respiratória, Transmissão de doença infecciosa

## Abstract

**Objective:**

To identify and synthesize scientific evidence that the use of face protection masks by children, in the community and at home, is a way of preventing communicable diseases.

**Data source:**

A scoping review was made using the Joana Briggs Institute method and PRISMA-ScR. A research was carried out in five electronic databases, at the Cochrane Library and on seven websites of governmental and non-governmental institutions. The data were organized in a spreadsheet and submitted to narrative analysis.

**Data synthesis:**

Initially, 658 productions were identified, of which 19 made up the final sample. Studies with higher levels of evidence are scarce. The types of masks identified were professional (surgical and facial respirators with filtration) and non-professional (homemade). The transmissible agents studied were influenza and SARS-CoV-2 viruses, and the evaluated environments were schools, homes and community spaces. The main discomforts reported were heat, shortness of breath, headache and maladjustment to the face. The indication and acceptability of masks change according to the age group and clinical conditions. There is no consensus on the reduction in the transmissibility of infections.

**Conclusions:**

Children older than five can benefit from the correct use of masks, as long as they are supervised, taught and educated to do so and the masks should be well adjusted to the face. The use of masks show better results when associated with other measures such as physical distancing, keeping places ventilated and frequent hand hygiene.

## RESUMO

**Objetivo:**

Identificar e sintetizar evidências científicas sobre o uso de máscaras faciais de proteção por crianças, na comunidade e no domicílio, como forma de prevenção de doenças transmissíveis.

**Fontes de dados:**

Revisão de escopo seguindo o método *Joana Briggs Institute* e o PRISMA-ScR. Realizou-se pesquisa em cinco bases de dados eletrônicas, na Biblioteca Cochrane e em sete sites de instituições governamentais e não governamentais. Os dados foram organizados em planilha e submetidos à análise narrativa.

**Síntese dos dados:**

Inicialmente foram identificadas 658 produções, das quais 19 compuseram a amostra final. Estudos com melhores níveis de evidência são escassos. Os tipos de máscaras identificadas foram as profissionais (cirúrgicas e respiradores faciais com filtragem) e as não profissionais (caseiras). Os agentes transmissíveis estudados foram os vírus influenza e SARS-CoV-2, e os ambientes avaliados foram escolas, domicílios e espaços comunitários. Os principais desconfortos relatados foram calor, falta de ar, dor de cabeça e desajuste ao rosto. A indicação e a aceitabilidade mudam conforme faixa etária e condições clínicas. Não há consenso sobre a redução da transmissibilidade de infecções.

**Conclusões:**

Crianças maiores de 5 anos podem se beneficiar com o uso correto de máscaras, desde que sejam supervisionadas e educadas para isso e que a máscara esteja bem ajustada ao rosto. Há melhores resultados quando seu uso está associado a outras medidas, como distanciamento físico, permanência em locais arejados e higiene frequente das mãos.

## INTRODUCTION

In the current global scenario of health emergency related to COVID-19, a clinical disease caused by the new coronavirus (SARS-CoV-2),^1^ which, until July 13^th^, 2021, affected about 180 million people worldwide and led to the death of more than 4 million,^2^ several prevention strategies have been recommended and adopted by different countries, including the use of protective masks by the population, including children.^3–5^

Although there is a relatively smaller proportion of COVID-19 cases in children, representing 1 to 5% of diagnosed cases, this group can develop severe forms of the disease. As of March 1^st^, 2021, 8,774 children up to 5 years of age were hospitalized in Brazil due to severe acute respiratory syndrome by COVID-19, of which 627 died. Mortality was more expressive among children up to one year of age, totaling 420 lives lost.^6^

The World Health Organization (WHO) recommends the use of face protection masks by the entire population as part of a comprehensive approach to reduce the transmission of SARS-CoV-2.^7^ Face masks cover both nose and mouth and may have a protective effect in blocking droplets and, if properly fitted on the wearer’s face, control air output.^3^ There are different types of masks available that provide different levels of protection, including filtering facepieces (PFF2 and N95), professional (surgical or procedural), and non-professional (homemade or fabric) masks.^7^

Among the benefits of the use of non-professional face masks by the general population is the possibility of reducing the risk of exposure to infectious agents by healthy people and of dispersion of respiratory particles by asymptomatic and symptomatic people. The disadvantages associated with the use of masks consist of a false sense of security and lower adherence to other preventive measures, the risk of self-contamination due to incorrect handling, the need for frequent changes and maintenance of hygiene, as well as headaches and respiratory discomfort. The difficulty of proper use by specific groups, such as younger children, and inappropriate disposal also prove to be challenges for the effectiveness of this prevention strategy.^7^ In this sense, some guidelines do not recommend the use of masks by children under 2 years of age, and the WHO also does not indicate the use by asymptomatic children aged between 2 and 5 years.^8^ On the other hand, some non-governmental scientific institutions, such as the Brazilian Society of Pediatrics and the American Academy of Pediatrics, issued a warning about the use of masks by children and adolescents, recognizing that this equipment can reduce the spread of secretions from sick or contaminated people and protect the community from particles suspended in the air, justifying its use as a preventive measure in childhood.^4,5^

There is still a scarcity of scientific studies with a high level of evidence on the benefit of the use of masks by children.^7,9^ In the Brazilian context, in an initial analysis, no consistent results were found on the use of masks by children as a way of preventing transmissible diseases. In addition, most international studies have a low level of scientific evidence.^7,10,11^ This gap makes it difficult to propose protocols and guidance to support the decision of managers and health professionals in instructing the population.

Given the context of the COVID-19 pandemic, it is relevant to generate evidence that can support original studies and synthesize information on the use of masks by children, including a specific recommendation from the WHO to carry out research on this subject.^7^ Given the above, the objectives of this study were to identify and synthesize scientific evidence on the use of protective face masks by children, in the community and at home, as a way of preventing communicable diseases.

## METHOD

This is a Scoping Review study, according to the method proposed by the Joanna Briggs Institute (JBI). This systematic review method makes it possible to examine the available evidence when studies on a particular topic are unclear or there is no systematic information on the subject.^12^ The research protocol was registered in the Open Science Framework^13^ platform and developed as recommended by the international guide of the Preferred Reporting Items for Systematic reviews and Meta-Analyses extension for Scoping Reviews (PRISMA-ScR).^14^

The review consisted of five steps: research identification; identification of relevant studies; selection of studies; data analysis; and data grouping, synthesis, and presentation.

For the elaboration of the research question, the mnemonic strategy PCC (participants, concept, and context) was used, in which P (participants) corresponds to children; C (concept), to face masks to protect against communicable diseases; and C (context), to community and domestic environments. Considering the above, the following question was elaborated: “What is the available scientific evidence about the use of masks by children, as a way of preventing communicable diseases, in the context of the community and the home?”.

Ethical assessment was not necessary for this research, as it uses data published in the public domain.

Searches were carried out independently by two reviewers, in July and August 2020, without time frame, in the following databases: Cumulative Index to Nursing and Allied Health Literature (CINAHL), MEDLINE (via PubMed), Latin American and Caribbean Health Sciences Literature (LILACS), Scopus, Web of Science, and the Cochrane library, as well as a search in gray bibliography accessed on the following pages of governmental and non-governmental institutions: Brazilian Society of Pediatrics (*Sociedade Brasileira de Pediatria* – SBP), National Agency for Sanitary Surveillance (*Agência Nacional de Vigilância Sanitária* – Anvisa), Centers for Disease Control and Prevention (CDC), World Health Organization (WHO), Portuguese Society of Pediatrics (*Sociedade Portuguesa de Pediatria* – SPP), European Academy of Pediatrics (EAP) and American Academy of Pediatrics (AAP).

An initial search was carried out on the PubMed and CINAHL portals to identify the main descriptors used in studies that dealt with the topic of interest from the search mneumonic. For each database, a search strategy was adapted with a similar combination of descriptors and keywords: “Mask”, “Children”, “Child”, “Communicable diseases”, “Communicable diseases control”, “Communicable diseases prevention”, “Health Personnel”, and the Booleans “and”, “or” and “not”. Different combinations of Booleans were used in each search due to the characteristics and number of productions in each database. Table 1 presents the database search strategy.

**Table 1:** Database search strategies.

Database	Search strategy
PubMed/MEDLINE	Respiratory Tract Infections OR Communicable diseases OR Communicable diseases control OR Communicable diseases prevention OR Coronavirus Infections AND Masks AND child AND children NOT Health Personnel
LILACS	Doenças transmissíveis OR controle de doenças transmissíveis OR infecções por coronavirus OR doenças respiratórias AND máscaras OR equipamento de proteção individual OR dispositivos de proteção respiratória AND criança OR pré-escolar.
Scopus	Mask AND Children OR Child AND Communicable Diseases
Cochrane Central	Mask AND Children OR Child AND Communicable Diseases NOT Health Personnel
*Web of Science*	Mask AND Children AND Communicable Diseases NOT Health Personnel
CINAHL	Mask AND Children AND Communicable Diseases NOT Health Personnel
SBP; Anvisa; SPP	Máscaras
CDC	Facemasks; Cloth face coverings; child
EAP AAP	Mask; Facemasks
WHO	Use of masks for children

Source: prepared by the authors. LILACS: Latin American and Caribbean Health Sciences Literature; CINAHL: Cumulative Index to Nursing and Allied Health Literature; SBP: Brazilian Society of Pediatrics; Anvisa: National Agency for Sanitary Surveillance; SPP: Portuguese Society of Pediatrics; CDC: Centers for Disease Control and Prevention; EAP: European Academy of Pediatrics; AAP: American Academy of Pediatrics; WHO: World Health Organization.

Inclusion criteria were: full texts of research published in full in English, Spanish, and Portuguese; that addressed the use of face masks by children under 18 years of age,^8^ as a way of preventing communicable diseases in the community and at home. Editorials, letters and theoretical essays, duplicate studies and studies that did not answer the research question were excluded.

Titles and abstracts were retrieved from the search and organized in a Microsoft Excel 2013 spreadsheet. All identified studies were evaluated, based on the established inclusion and exclusion criteria, by two researchers (PPB and MSS) independently, to identify potentially eligible ones. When there was no consensus among researchers, the article was kept in the database for the next phase, which involved reading each of the selected articles in full in order to confirm their relevance and, if so, extract the data. Inconsistencies or doubts were resolved by consensus among the authors.

The articles selected according to eligibility were read and reread by the researchers and constituted the final sample. Titles, type of study, year of publication, level of evidence according to the JBI classification,^12^ country of origin, objective, methodology, sample, communicable disease, types of masks, context and results of articles and documents were organized into a Microsoft Excel 2013 software spreadsheet. This was followed by the narrative analysis of the data with subsequent discussion of the results with the relevant bibliography.

## RESULTS AND DISCUSSION

**Figure 1.**
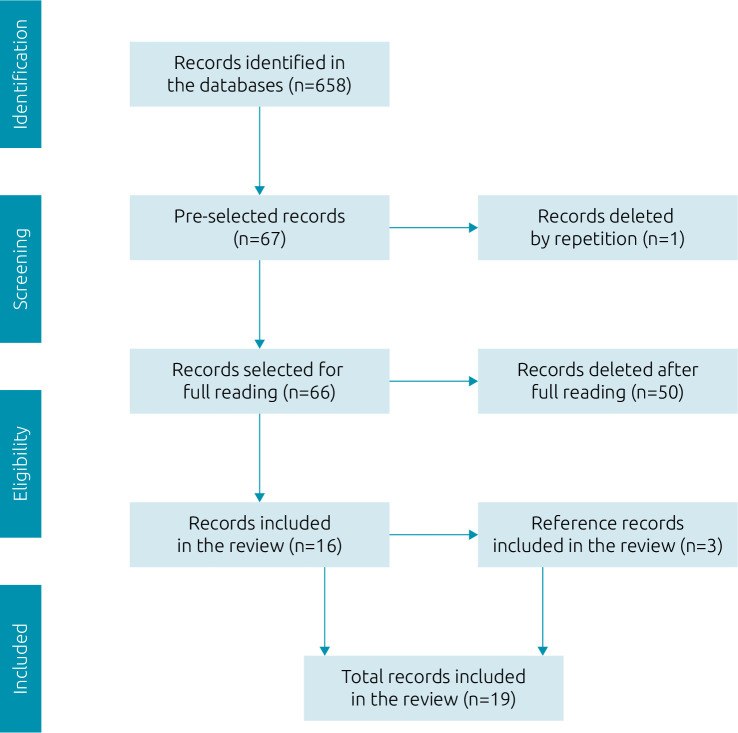
Flowchart of the review studies selection process, adapted from PRISMA-ScR. Brazil, 2020.

Source: prepared by the authors.

The initial search generated a total of 658 studies. After examining the title and abstract, 66 works were selected for full reading, 39 articles and 27 documents. After reading in full, 50 publications were excluded for not answering the research question, leaving nine articles and seven documents. The reference lists of these studies were analyzed and, from that, it was possible to include three more articles that answered the research question. Thus, 19 productions made up the final sample of this review. Figure 1 represents the selection flow of the materials found, according to PRISMA-ScR guidelines.

Among the studies that composed this review, eight (42.1%) were published between 2008 and 2017 — all of these productions evaluated the topic of influenza infection. On the other hand, 11 productions (57.9%) were made in 2020, all of which referring to the context of the COVID-19 pandemic. Regarding the origin of the productions, six (31.5%) were developed in Asian countries, five (26.3%) in Europe, and eight (42.2%) in America (Table 2).

**Table 2:** Characterization of the manuscripts included in the review.

Study design	LE	Sample	Country	Type of mask	Communicable disease	Year
Randomized clinical essay^15^	1c	221 (<6 years) and 163 (7 to 11 years)	Thailand	Surgical	Influenza	2011
Observational^16^	4b	8,569 (6 to 13 years)	China	NI	COVID-19	2020
Observational^17^	4b	503 (preschoolers and schoolchildren)	United States	NI	Influenza	2010
Randomized clinical essay^18^	3c	39 (mean age of 7 to 9 years)	Germany	Surgical	Influenza	2011
Observational^19^	4b	494 students (6 to 12 years)	China	NI	Influenza	2008
Non-randomized clinical essay^20^	2c	11 (5 to 11 years)	Netherlands	Surgical PFF2 Homemade	Influenza	2008
Observational^21^	4b	10,524 (7 to 12 years)	Japan	NI	Influenza	2017
Randomized clinical essay^22^	2c	88 (<5 years) and 232 (6 to 15 years)	China	Surgical	Influenza	2009
Opinion article^23^	5b	NA	China	Surgical PFF2 Homemade	COVID-19	2020
Opinion article^24^	5b	NA	Italy	NI	COVID-19	2020
Opinion article^25^	5b	NA	Canada	NI	Influenza	2010
Opinion article^26^	5b	NA	Italy	Surgical PFF2	COVID-19	2020
**Type of document**		**Institution**	**Country**	**Mask**	**Disease**	**Year**
Recomendations^8^	5b	OMS	Switzerland	Homemade PFF2	COVID-19	2020
Recomendations^27^	5b	AAP	United States	Homemade PFF2	COVID-19	2020
Recomendations^28^	5b	AAP	United States	Homemade	COVID-19	2020
Recomendations^29^	5b	AAP	United States	Homemade	COVID-19	2020
Recomendations^29^	5b	AAP	United States	Homemade PFF2	COVID-19	2020
Guide^30^	5b	CDC	United States	Homemade	COVID-19	2020
Note of alert^4^	5b	SBP	Brazil	Homemade PFF2	COVID-19	2020

LE: level of evidence:^31^ 1c: randomized clinical essays; 2c: almost experimental; 3c: cohort with control group; 4b: cross-sectional; 5b: expert consensus; NI: not informed; PFF2: filtering facepiece 94%; NA: not applicable; WHO: World Health Organization; AAP: American Academy of Pediatrics; CDC: Centers for Disease Control and Prevention; SBP: Brazilian Society of Pedicatrics.

Regarding the population studied, six productions (31.5%)^15–17,22^ developed the study on the use of masks including children and adults (teachers and family members), and two (10.5%)^16,21^ were performed exclusively with children. The other productions, which dealt exclusively with children,^4,5,8,23–30^ did not carry out field research, as they were articles or documents that presented opinions or recommendations from experts, being classified by the JBI as level of evidence 5.^31^ Six (31.5%) studies analyzed the use of masks concomitantly with other preventive measures, such as hand hygiene,^15,16,18,22^ use of hand sanitizer/alcohol^17^, and staying in an airy environment^19^ (Table 3).

**Table 3:** Main results and environmental context of the manuscripts included in the review.

Main results (scientific articles)	Context
Influenza transmission was not reduced by wearing a face mask and washing hands.^15^	Household
32.47% of children wore properly fitted masks, 42.42% had difficult access to children’s masks. The higher the mother’s education, the better the child’s behavior wearing them.^16^	Community environments
The regular use of the mask by children was 50%, it reduced over the days, caused distraction and physical discomfort, made communication between students and teachers difficult, and it was difficult to adjust to the face.^17^	School
There was no significant difference in adherence and in the use of masks by children and adults. Most did not report problems with use, but there were discomforts. Mask use was influenced more by intervention than by the participants’ perception of its importance.^18^	Household
The use of a mask was not enough to contain the potential for the spread of influenza. However, a reduction in the number of infections was identified when prevention measures (vaccination, use of mask, and well-ventilated environment) were associated.^19^	School
All types of masks provide protection by reducing particulate exposure during activities. Protection of children was lower than that of adults. PFF2 masks offered ten times greater protection than homemade masks and six times greater protection than surgical ones.^20^	Community Environment
Mask use showed a significant protective association for influenza infection, varying between child age groups.^21^	Everyday
With handwashing and masking intervention, the risk of influenza virus infection was significantly higher for children aged 6 to 15 years and for household contacts with children who were the index case.^22^	Household
Children over 2 years of age must wear a mask, adapted to the face, associated with other preventive measures. N95 masks should only be used by high-risk children. Parental guidance should be carried out.^23^	Community environments
Children over 2 years of age must wear a mask fitted to the face. In preschoolers, mask use can only be achieved through thorough work by parents or guardians. N95 should only be used by high-risk children.^24^	Community environments
The use of face masks can be an effective, practical intervention and a non-pharmacological strategy to reduce the spread of influenza.^25^	School
Children over 2 years of age must wear a mask in crowded environments. Parents or guardians must educate children on correct handling. Do not use masks for the practice of physical activities. The first choice is the surgical mask; N95 only for high-risk children.^26^	Community environments
Main results (documents)	Context
Children under 5 years of age should not wear a mask. Between 6 and 11 years, use under adult supervision and in association with frequent hand washing, physical distancing, and staying in well-ventilated places.^8^	Community environments
The use of a mask by children with special needs should be carried out in environments where distancing is not possible and under professional guidance.^27^	Community environments
Children over 2 years of age can wear a mask. Children with special conditions should receive extra attention.^28^	School
During the practice of physical activities, children should wear the mask when distancing is not possible. Do not use it during aquatic activities and when there is a risk of accident with the mask.^29^	Physical activity
Children over 2 years of age must wear a mask adapted to the face. Parents or guardians must educate children about its use. The use of protectors as a substitute for masks is not recommended.^5^	School
Children over 2 years of age should wear the mask when it is not possible to keep the distance. Children at risk should use the N95 model and family members, the surgical one.^30^	Community environments
Children over 2 years of age must wear a mask and parents or guardians must educate children about its correct handling. For children with special conditions, the need must be assessed.^4^	Community environments

PFF2: filtering facepiece.

The present review allowed us to identify scientific evidence about the use of masks by children, in the prevention of transmission of influenza and SARS-CoV-2 infection, indicating that there are specificities, depending on the age group, the environment, and the association with other protection measures. Eight productions identified do not recommend the use of masks by children under 2 years of age, with the justification that there is an increased risk of suffocation.^5,23–25^

Three original studies^15,17,22^ included children aged between 2 and 5 years in their samples, and identified that adherence to the regular use of masks in the school environment was reduced as the days went by, with disuse factors being distraction, physical discomfort, and difficulty in communication between the child and the teachers.^17^ In addition, in the home environment, two of these studies showed that, together with the habit of frequent hand hygiene, the use of a mask by children with Influenza did not reduce significantly the risk of infection in the family.^15,22^

In preschool children, efficient mask use can only be achieved through a thorough work with parents or guardians.^23^ Due to the low understanding of the need for mask use, child adherence is sometimes so poor that it is better to stop wearing a mask and just adopt other preventive measures, such as social distancing and frequent hand hygiene.^24^ Three studies with a low level of scientific evidence^23,25,26^ and four documents from governmental^30^ and non-governmental^5,28,29^ institutions stated that children over 2 years of age should wear a face mask as a way of preventing contamination and the spread of infectious diseases, especially in school environments. On the other hand, the WHO does not recommend the use of masks by asymptomatic children aged between 2 and 5 years, considering the scarcity of consistent evidence on the subject, its potential interference in child development and the low ability and autonomy this age group has to use them correctly. In this sense, the WHO states that, in strictly necessary situations, the use of a mask by children in this age group must be systematically supervised by an adult.^8^

The analysis of the articles’ content allows us to infer that there is a lack of consensus on the use of masks, especially for children under 5 years of age. It was identified that this happens due to the scarcity of robust or clinical studies for these age groups. This scarcity has not allowed the production of consensual responses by governmental and non-governmental bodies, and they end up providing guidance on the use of masks in childhood based on the opinion of experts who are part of these institutions.

The analysis of the publications confirms that the results involving the evaluation of the protective effect of the use of masks by children under 5 years old are scarce and inconsistent, and that more rigorous and comprehensive studies need to be developed to support managers and health professionals in decisions regarding its use in children in this age group. In addition, the WHO suggests that studies involving the use of masks in childhood should also take into account school environments frequented by low- and middle-income populations, due to existing social and health inequalities, in order to create assistance policies to minimize them.^8^

Studies involving children over 5 years of age presented mixed results. A study carried out in Wuhan, China,^16^ with 8,569 children aged 6 to 13 years old, showed that only 32% of children wore properly adjusted masks and that the higher the mother’s education, the better the child’s behavior wearing them. This study also found that 42% of the participants said it was difficult to buy children’s masks that fit properly to the face.^16^ Several studies indicate that children in this age group can be educated and guided by their parents or guardians to use them,^4,8,18,23,26^ with evidence that children, even when sick, have acceptability and adherence comparable to those of adults, when well oriented.^18^ The use of a face mask and frequent hand hygiene have better results when properly instructed by parents, guardians, and educators, including demonstrations of how to use and what care should be taken when putting on, keeping, and removing the mask.^18,23^ However, the main discomforts described by children in the studies include headache,^18^ shortness of breath,^18^ feeling of heat,^17,18^ and inadequate fit to the face.^17^

The analysis of the productions makes it possible to identify that, for better results regarding the use of masks, additional preventive measures should be adopted, such as frequent hand hygiene,^8,18,19,22,23,27,28^ physical distancing,^8,23,29^ and permanence in ventilated places.^8,19^ A study conducted in a school environment showed that the use of masks with a filtration efficiency of 80%, associated with better environmental ventilation, was not enough to contain the potential for the spread of the influenza virus among schoolchildren. However, the results indicated a reduction in the number of infections when multiple prevention measures (vaccination, mask use, and ventilated environment) were associated.^19^ In contrast, a Japanese study with 10,524 children aged 7 to 12 years found that the use of mask showed a significant protective association against influenza infection.^21^

It is necessary to consider that the use of masks involves cultural issues and that preparing the child to wear them is extremely necessary,^23,24^ in addition to recognizing that encouraging the use of masks, concomitantly with other preventive measures,^8,18,19,22,23,27–29^ seems to be a timely and valid alternative for a scenario of uncertainties such as the current pandemic situation caused by COVID-19. However, it should be known that adopting these measures properly has not been a simple task. This is evidenced in a meta-analysis study dedicated to identifying interventions that generate personal behavior change (use of face mask, hand hygiene, disinfection of surfaces, physical distancing, among others) to limit the spread of respiratory viruses in adults and children.^32^ This study highlights that just guiding and advising people has been insufficient.^32^

These findings expand what was found in the present scoping review and indicate that the use of masks by the general population, including children, even when other prevention measures are adopted, requires a systematic process of guidance, health education, involvement, and evaluation regarding the measures at matter.

In this sense, it is noteworthy to consider that strategies such as health literacy can favor the process of adherence to measures to reduce the spread of respiratory viruses, such as SARS-CoV-2. Health literacy comprises the cognitive and social skills that determine an individual’s motivation and ability to access, understand, and use information in ways that promote and maintain good health.^33^

A multiple case study, developed in Hong Kong, which aimed to explore the health literacy of Chinese parents with preschool-age children, in relation to the prevention of seasonal influenza in childhood, highlights that the health literacy process allows individuals to exercise a degree of autonomy and security for decision-making that, in turn, contribute to the prevention of this viral disease.^34^ In this sense, it is possible to recognize that the health literacy strategy for the family context on the concomitant use of preventive measures, including the proper use of masks in childhood, may contribute to reducing the spread of respiratory infections.

This review identified four studies^16,17,19,21^ that did not specify the type of mask used by children. However, another study found that all types of masks provide protection, reducing exposure to particles during activities for both children and adults, but with greater efficiency in adults, which may be related to the inadequate fit of the mask on children’s faces.^20^ In any case, PFF2 masks (filtering facepiece 94%) provided ten times greater protection than homemade masks and six times greater protection than surgical masks.^20^ Another study pointed to the surgical mask as the type of first choice for the child population.^26^

For children with greater vulnerability, including those with chronic or immunosuppressed conditions, some publications recommend the use of an N95 or PFF2 mask, under the guidance of health professionals, who can assess its real need and proper fit to the face.^4,5,23,26,28,30^ On the other hand, other documents state that the use of masks by children and adolescents with special needs should only occur in environments where physical distancing is not possible, individually and based on professional guidance, with planning and observing the child’s acceptance.^27^

Another study warns that the available N95 mask has not been validated for use in younger children, and the larger size of the device may lead to a loss of effectiveness. However, the authors acknowledge that when pediatric-approved masks are available, children with severe chronic conditions may benefit from their use.^24^

In situations involving sports, the American Academy of Pediatrics recommends that children wear a cloth mask when it is not possible to maintain a safe distance, except in water sports and other sports that may pose a risk of injury from being trapped in the equipment or accidentally covering the eyes.^29^ Another study^26^, in turn, does not recommend the use of face masks during sports activities.

This review identified that, in the context of the COVID-19 pandemic, the real benefit of the use of masks by the pediatric population, as well as the types of masks that should be used in healthy individuals or those with severe chronic disease, cannot be definitively established.^24^

The studies analyzed in this Scoping Review showed that there is no scientific definition of the role of the use of masks by children in the prevention of communicable infectious respiratory diseases. The low level of scientific evidence signals the urgent need to move forward with experimental clinical studies that can elucidate the problem raised.

However, the children’s self-care ability, whether according to age or their ability to follow recommendations, seems to influence both the way in which this protective equipment is used and the results achieved. Thus, children over 5 years of age can benefit, especially in crowded environments, with the correct use of masks, reducing the chances of contamination and spread of these diseases, as long as they are properly educated, guided, and supervised.

As limitations of this study, it is possible that there are productions available in databases that have not been addressed, despite the fact that publications were sought in robust and reputable databases. Furthermore, most of the included studies have a low level of scientific evidence, which did not contribute to the generalization of the results.

Proper mask use appears to have better results when combined with other preventive measures, including frequent hand hygiene, physical distancing, and staying in ventilated areas. However, it is a challenge to find face masks with an adequate fit to the child’s face, in addition to the discomfort generated by their use, especially in younger children, with few studies evaluating this issue.

It is recommended that experimental clinical studies can be dedicated to studying not only the behavior of children of different ages regarding the use of masks, but also revealing the cost and benefit of this use, including a comprehensive analysis regarding the effectiveness of different types of masks, their impacts on child development, safety measures, and indications and contraindications for their use.

## References

[1] World Health Organization [homepage on the internet] (2020). Novel Coronavirus (2019-nCoV) Situation Report – 22.

[2] Johns Hopkins University & Medicine [homepage on the internet] The Johns Hopkins 30-Minute COVID-19 Briefing: expert insights on what you need to know now.

[3] Brazil – Ministério da Saúde (2020). Agência Nacional de Vigilância Sanitária [homepage on the internet]. Orientações gerais: máscaras faciais de uso não profissionais.

[4] Sociedade Brasileira de Pediatria [homepage on the internet] (2020). Uso de máscaras faciais em tempo de COVID-19 por crianças e adolescentes: uma proposta inicial.

[5] American Academy of Pediatrics (2020). Cloth face coverings for children during COVID-19 [homepage on the internet].

[6] Brazil - Ministério da Saúde (2021). Boletim Epidemiológico Especial 52 [homepage on the internet].

[7] World Health Organization (2020). Interim guidance.

[8] World Health Organization [homepage on the internet] (2020). Advice on the use of masks for children in the community in the context of COVID-19. Annex to the Advice on the use of masks in the context of COVID-19.

[9] Huang FS, Schaffzin JK (2021). Rewriting the playbook: infection prevention practices to mitigate nosocomial severe acute respiratory syndrome coronavirus 2 transmission. Curr Opin Pediatr..

[10] Jefferson T, Del Mar CB, Dooley L, Ferroni E, Al-Ansary LA, Bawazeer GA (2010). Physical interventions to interrupt or reduce the spread of respiratory viroses. Cochrane Database Syst Rev..

[11] Taminato M, Mizusaki IA, Saconato H, Franco ES, Puga ME, Duarte ML (2020). Homemade cloth face masks as a barrier against respiratory droplets - systematic review. Acta Paul Enferm..

[12] Joanna Briggs Institute [homepage on the internet] (2017). The JBI Approach. Grades of recommendation. Levels of Evidence.

[13] Braga P, Oliveira PP, Romano MC, Gesteira EC, Souza M Use of masks by children for the prevention of transmissible diseases: scoping Review.

[14] Tricco AC, Lillie E, Zarin W, O’Brien KK, Colquhoun H, Levac D (2018). PRISMA Extension for Scoping Reviews (PRISMA-ScR): checklist and Explanation. Ann Intern Med..

[15] Simmerman JM, Suntarattiwong P, Levy J, Jarman RG, Kaewchana S, Gibbons RV (2011). Findings from a household randomized controlled trial of hand washing and face masks to reduce influenza transmission in Bangkok, Thailand. Influenza Other Respir Viruses..

[16] Chen X, Ran L, Liu Q, Hu Q, Du X, Tan X (2020). Hand hygiene, mask-wearing behaviors and its associated factors during the COVID-19 Epidemic: a cross-sectional study among primary school students in Wuhan, China. Int J Environ Res Public Health..

[17] Allison MA, Guest-Warnick G, Nelson D, Pavia AT, Srivastava R, Gesteland PH (2010). Feasibility of elementary school children’s use of hand gel and facemasks during influenza season. Influenza Other Respir Viruses..

[18] Suess T, Remschmidt C, Schink S, Luchtenberg M, Haas W, Krause G (2011). Facemasks and intensified hand hygiene in a German household trial during the 2009/2010 influenza A(H1N1) pandemic: adherence and tolerability in children and adults. Epidemiol Infect..

[19] Chen SC, Liao CM (2008). Modelling control measures to reduce the impact of pandemic influenza among schoolchildren. Epidemiol Infect..

[20] van der Sande M, Teunis P, Sabel R (2008). Professional and home-made face masks reduce exposure to respiratory infections among the general population. PLoS One..

[21] Uchida M, Kaneko M, Hidaka Y, Yamamoto H, Honda T, Takeuchi S (2016). Effectiveness of vaccination and wearing masks on seasonal influenza in Matsumoto City, Japan, in the 2014/2015 season: an observational study among all elementary schoolchildren. Prev Med Rep..

[22] Cowling BJ, Chan KH, Fang VJ, Cheng CK, Fung RO, Wai W (2009). Facemasks and hand hygiene to prevent influenza transmission in households: a randomized trial. Ann Intern Med..

[23] Esposito S, Principi N (2020). To mask or no to mask children to overcome COVID-19. Eur J Pediatr..

[24] Esposito S, Principi N (2020). Mask-wearing in pediatric age. Eur J Pediatr..

[25] Del Valle SY, Tellier R, Settles GS, Tang JW (2010). Can we reduce the spread of influenza in schools with face masks?. Am J Infect Control..

[26] Jin K, Min J, Jin X, Re: Esposito (2020). To mask or not to mask children to overcome COVID-19. Eur J Pediatr..

[27] American Academy of Pediatrics [homepage on the internet] (2020). COVID-19: caring for children and youth with special health care needs.

[28] American Academy of Pediatrics [homepage on the internet] (2020). Return to school during COVID-19.

[29] American Academy of Pediatrics [homepage on the internet] (2020). Kids and masks: why cloth face coverings are needed in youth sports during COVID-19.

[30] Centers for Disease Control and Prevention [homepage on the internet] (2020). Operating schools during COVID-19: CDC’s considerations.

[31] Joanna Briggs Institute [homepage on the internet] (October 2013). JBI Levels of Evidence. Developed by the Joanna Briggs Institute Levels of Evidence and Grades of Recommendation Working Party.

[32] Perski O, Szinay D, Corker E, Shahab L, West R, Michie S (2022). Interventions to increase personal protective behaviors to limit the spread of respiratory viruses: a rapid evidence review and meta-analysis. Br J Health Psychol..

[33] World Health Organization (2015). Health literacy and health behavior 2015.

[34] Lam W, Dawson A, Fowler C (2015). The health literacy of Hong Kong Chinese parents with preschool children in seasonal influenza prevention: a multiple case study at household level. PLoS One..

